# Nutrition: From Bench to Bedside

**DOI:** 10.1155/2016/4295179

**Published:** 2016-08-10

**Authors:** Naim A. Khan, Azeddine Ibrahimi, Aziz Hichami, Najat Mokhtar

**Affiliations:** ^1^INSERM U866, Université Bourgogne-Franche-Comté, 2100 Dijon, France; ^2^Medical Biotechnology Lab, Faculty of Medicine and Pharmacy, Mohammed V University at Souissi, 10000 Rabat, Morocco; ^3^INSERM U866, Cancer Chemotherapy Group, Université Bourgogne-Franche-Comté, 2100 Dijon, France; ^4^Nutrition and Health Section, International Atomic Energy Agency, 1400 Vienna, Austria

The nutritional health in the world is one of the most pressing issues facing different countries today. International organizations like FAO and WHO suggest that there is an improvement of the nutritional situation in Asia and Latin America, though a deterioration in Sub-Saharan Africa does exist. As regards Europe and USA, we are living in the era of “overnutrition.” Unfortunately, the situations of famine, hunger, and starvation do exist in some developing countries. Some of the noncommunicable pathologies like obesity are not only due to the excess of fatty food, but intake of unbalanced diet also contributes significantly to their pathogenesis. Nutrient requirements vary as a function of lifestyle. Infants and pregnancy require more attention because these situations are more vulnerable and are at greater risk for malnutrition that might contribute to metabolic memory, responsible for the pathologies like macrosomia, obesity, type 2 diabetes, and hypertension. To cover all of these areas, there is a pressing need to develop a platform for the translational medicine from fundamental to clinical research in the field of nutrition.

This special issue was initially destined to publish the peer-reviewed and selected articles that were presented in the 1st International Congress of Nutrition & Food Science https://www.univ-tlemcen.dz/, held in Tlemcen (Algeria) from 20th to 22nd November 2015; however, the issue was open to all other original and high-quality unpublished articles belonging to the topics of the issue.

The hot topics of the special issue included the following fields: phytotherapy, pediatric nutrition, food security, immunonutrition, pathophysiological complications and nutrition, clinical outcome and nutrition, experimental nutrition and nutrients, and nutritional assessment.

In this issue, we received a large number of articles, but we gave the priority to the high-quality manuscripts during the reviewing process. This issue is comprised of 8 articles which deals with different aspects of nutrition and its importance in health and disease. The phytotherapy has been increasingly considered as safe and an alternative to allopathic medicine which might cause the side effects in the long run. In this issue, two articles deal with plant-derived products in different pathophysiological states. Immune system, particularly the inflammatory state, contributed by high concentrations of proinflammatory cytokines, is the cause of several pathological manifestations. The n-3 polyunsaturated fatty acids (n-3 PUFA) have been proposed to exert beneficial effects during hypertension and dementia. Besides, these agents exert anti-inflammatory effects.

Two articles deal with the immunomodulatory/anti-inflammatory effects of vitamin D3 and n-3 PUFA. There are two articles that contribute to this burning topic. Micronutrition has been proposed as the remedy that exerts immediate beneficial effects as it fulfils the endogenous requirements of the body. Indeed, the deficiency of micronutrients might be the cause of different pathologies. Two articles deal with this subject where one of them sheds light on the role of glutamine and the second one plausibly demonstrates that the deficiency of iron in the primary school children might be associated with their reduced learning capacity and supplementation of iron in their diet improves this parameter.

